# *Staphylococcus aureus* biofilm: Formulation, regulatory, and emerging natural products-derived therapeutics

**DOI:** 10.1016/j.bioflm.2023.100175

**Published:** 2024-01-01

**Authors:** Xiying Wu, Huan Wang, Juan Xiong, Guo-Xun Yang, Jin-Feng Hu, Quangang Zhu, Zhongjian Chen

**Affiliations:** a Shanghai Skin Disease Hospital, Tongji University School of Medicine, Shanghai, 200443, China; b Institute of Natural Medicine and Health Products, School of Pharmaceutical Sciences, Zhejiang Provincial Key Laboratory of Plant Evolutionary Ecology and Conservation, Taizhou University, Zhejiang, 318000, China; c Department of Natural Medicine, School of Pharmacy, Fudan University, Shanghai, 201203, China; d School of Pharmacy, Naval Medical University, Shanghai, 200433, China

**Keywords:** Staphylococcus aureus, Biofilm, Antibiofilm strategies, Targets, Natural products

## Abstract

*Staphylococcus aureus* can readily form biofilm which enhances the drug-resistance, resulting in life-threatening infections involving different organs. Biofilm formation occurs due to a series of developmental events including bacterial adhesion, aggregation, biofilm maturation, and dispersion, which are controlled by multiple regulatory systems. Rapidly increasing research and development outcomes on natural products targeting *S. aureus* biofilm formation and/or regulation led to an emergent application of active phytochemicals and combinations. This review aimed at providing an in-depth understanding of biofilm formation and regulation mechanisms for *S. aureus*, outlining the most important antibiofilm strategies and potential targets of natural products, and summarizing the latest progress in combating *S. aureus* biofilm with plant-derived natural products. These findings provided further evidence for novel antibiofilm drugs research and clinical therapies.

## 1 Introduction

*Staphylococcus aureus* is a drug-resistant pathogen that can cause skin and soft tissue infections, further leading to severe endocarditis, osteomyelitis, pneumonia, and other invasive diseases [1]. In addition to the wide panel of secreted virulence factors, biofilm formation is a significant feature that promotes the development of drug-resistance for *S. aureus* [2]. It has been reported that biofilm can provide significant survival advantages to microbial communities, resulting in up to a 1500-fold increase in strain resistance [3]. Accordingly, developing new antibiofilm agents against *S. aureus* is of urgent importance.

Natural products have been extensively explored as important sources of biocompatible antibiofilm agents to avoid the side effects of traditional antibiotics on human health and the environment. For instance, essential oils from plants [4,5], flavonoids [6,7], phenolic acids [8], and terpenoids [9,10] showed biofilm inhibitory and disrupting activity through different mechanisms, including reducing adhesins, destroying biofilm matrix and interrupting bacterial communication. Additionally, an increasing number of new antibiofilm targets of natural products have been studied using molecular biology methods and computer virtual screening [11,12]. The obtained findings provide the basis for screening natural and effective antibiofilm alternatives.

In light of biofilm's complex and rapid adaptability, this review provided an in-depth understanding of biofilm formation and regulation mechanism for *S. aureus*, outlining the promising antibiofilm strategies and potential targets of natural products. We analyzed the recent advancements, further elucidated the innovative approaches based on plant-derived natural products to combat *S. aureus* biofilms, and pointed out new directions for future investigations. Combining plant-derived natural products with other antibacterial compounds or techniques to increase the activity was also discussed, adding to the design of novel therapeutics to fight against this clinically significant pathogen.

## 2 Formation and properties of *S. aureus* biofilm

**Fig. 1:**
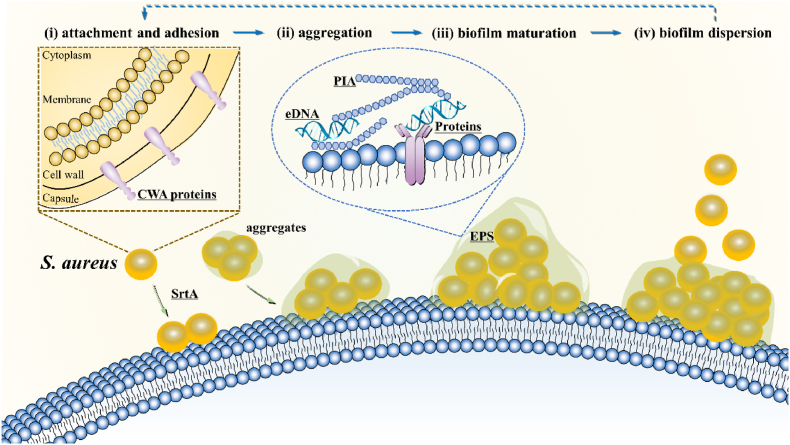
*S. aureus* biofilm formation process: (i) initial attachment and adhesion, in which single cells or aggregates adhere to surfaces; (ii) aggregation, with cell division and proliferation as well as EPS production; (iii) biofilm structuring and maturation, where microorganisms coexist within polymicrobial interactions; and (iv) biofilm dispersion, with cell detachment from the aggregate biofilm to planktonic state. EPS, extracellular polymeric substances; eDNA, extracellular DNA; PIA, Polysaccharide intercellular adhesin; CWA proteins, cell wall-anchored proteins; SrtA, sortase A. Key molecules of potential antibiofilm targets are underlined.

The biofilm development process can be didactically divided into the following four stages: (i) attachment and adhesion; (ii) aggregation with extracellular matrix synthesis and bacterial proliferation; (iii) biofilm structuring and maturation; and (iv) biofilm dispersion with cell detachment [13,14] (Fig. 1).

During the initial adhesion or aggregation stage, *S. aureus* planktonic cells attach to biotic or abiotic surfaces and are enclosed in complex covering composed of host macromolecules such as proteins [15,16]. During tissue infections, bacteria attach to each other, forming aggregates in viscous mucus (e.g., cystic fibrosis [17]) or on damaged host tissues (e.g., heart valves [18], bones [19], or skin of chronic wounds [20]).

During the maturation and proliferation stage, the attached or aggregated cells multiply and produce an extracellular polymeric substance (EPS), indispensable for constructing three-dimensional biofilm scaffolds [21]. EPS molecules, including polysaccharides, nucleic acids, proteins, and lipiden biofilm cells to provide mechanical stability for the biofilm, thus directly determining the living conditions of the cells [22].

In the biofilm diffusion stage, the biofilm is destabilized with cells and aggregates, dispersing into the environment, attaching to new locations and triggering new biofilm infections. New biofilms locations hinge on the transport opportunity of the detached bacteria, resulting in new infections in nearby tissues, such as implant biofilms causing osteomyelitis [23,24], or farther locations, such as detached biofilms causing endocarditis [25].

Bacterial cells divide and the matrix complexity increase, creating physiological heterogeneity inside the biofilm that is characterized mainly by gradients of nutrients and oxygen [26]. The cells growing within the biofilm are generally categorized into four metabolic states: (i) aerobic (located in the oxygenated and nutrient-rich outer layer); (ii) fermentative (located in the oxygen- and nutrient-poor inner layer); (iii) dormant (located in anoxic layer with slow growth and inactive metabolism); and (iv) dead [[27], [28], [29]]. Dormant cells can result in a drop in intracellular adenosine triphosphate, making bacteria less sensitive to antibiotics [30]. Besides, some other gradients are present in biofilms. For instance, a vertical gradient of viscoelasticity is established during the preliminary stages of *S. aureus* biofilm development, which also facilitates biofilm dispersal, removing loosely bound bacteria while retaining an entrenched layer in the biofilm structure [31]. Rivera et al. revealed stark lipidomic heterogeneity in *S. aureus* biofilm, demonstrating that each horizontal layer was molecularly distinct [32]. Future applications of these findings to study spatially localized molecular responses to antibiofilm agents could provide new therapeutic strategies.

## 3 Key molecules in *S. aureus* biofilm formation

The degradation of the EPS matrix is of particular relevance for antibiofilm measures. Thus far, various agents have been applied to remove single-species and mixed-population biofilms, primarily by degrading self-produced adhesins, nucleic acids, and polysaccharides [[33], [34], [35], [36]].

### 3.1 Polysaccharide intercellular adhesin (PIA)

PIA is the majority of EPS components for *S. aureus* biofilm, which has a vital role in several aspects, including colonization, biofilm formation, immune evasion, and antibiotic resistance [37,38]. PIA production is strongly dependent on the environmental conditions such as anaerobiosis and glucose [37] and controlled by different regulatory systems [39]. Over the years, a variety of regulatory proteins and genes have been found to regulate *ica* expression, which might underlie the differential expression of PIA in different staphylococcal strains [40]. The PIA-dependent biofilm is predominantly observed in methicillin-sensitive *S. aureus* strain [37,38]. In this mechanism, PIA makes the polymer process a net positive charge through deacetylation to complete cell attachment and intercellular adhesion, increasing biofilm retention and drug resistance [41]. Biochanin A is a natural isoflavonoid that can prevent biofilm formation by inhibiting the release of PIA and disintegrating the preformed biofilms by dissociated EPS matrix [42]. Furthermore, the natural (+)-nootkatone significantly prevented the *S. aureus* biofilm formation with an inhibition rate of > 90 % at 50 μg/mL by depressing the *icaA* expression [43]. Moreover, PIA connects to various proteins with different effects. For example, biofilm-associated protein (Bap) cooperates with PIA to promote cell-to-cell aggregation during the process of biofilm formation [44], and PIA interacts with the accumulation-associated proteins (Aap) to promote the maturation of staphylococcal biofilm [45]. Therefore, *ica* operon and proteins such as Aap and Bap might be considered potential targets in the PIA-dependent biofilm.

### 3.2 Extracellular proteins

At the beginning of biofilm formation, adhesion to the surfaces is the main strategy for *S. aureus* cells to invade host cells [46]. In this process, *S. aureus* expresses a large number of surface proteins covalently linked to peptidoglycans, also known as cell wall-anchored (CWA) proteins. Microbial surface components recognizing adhesive matrix molecules (MSCRAMMs), the most prevalent CWA proteins, include protein A, fibronectin-binding proteins (FnBPs), clumping factors (ClfA, ClfB), serine-aspartate repeat family proteins (SdrC, SdrD, and SdrE), biofilm-associated protein (Bap), fibrinogen-binding protein (Fib), and *S. aureus* surface protein (SasG) [47,48]. They facilitate the adhesion of *S. aureus* cells to EPS and the host cells, thus promoting biofilms accumulation [49]. Natural products, such as luteolin, quercetin, and kaempferol from *Euphorbia humifusa* have been reported to suppress biofilm formation by down-regulating these surface adhesion proteins encoding genes (e.g., *fnbpA*, *fnbpB*) [50].

Transpeptidation mediated by membrane sortase A (SrtA) is essential for anchoring CWA proteins to the cell wall envelope. Knockout of SrtA could inhibit the assembly of surface adhesins in the cell wall envelope and the impact of acute infection [51,52]. Many natural products have been investigated as inhibitors of SrtA. For instance, kaempferol was found to inhibit SrtA activity and down-regulate the expression of CWA proteins-related genes, thus inhibiting the formation of *S. aureus* biofilm [53]. Likewise, chalcone, quercetin, and myricetin were also found to exert effective inhibitory activity against SrtA [54,55]. Moreover, molecular dynamics simulations and mutagenesis assays of chalcone against SrtA implied that the inhibitory activity lies in the interactions between chalcone and SrtA residues Val168, Ile182, and Arg197 [55]. Therefore, interference with surface proteins anchoring by targeting the corresponding genes or SrtA and modulating the extracellular enzymes by targeting the global regulators are promising antibiofilm strategies to combat *S. aureus* infections.

### 3.3 Extracellular DNA (eDNA)

eDNA exerts various biological functions, including adhesion, gene transfer, and DNA damage repair [56,57]. Besides, in *Pseudomonas aeruginosa* biofilms, eDNA cross-linking with lipoprotein anchored in the membrane can connect the matrix to microbial cells within the biofilm and protect against enzymatic attack from DNase [58]. Moreover, the holin-like protein (CidA) was indicated to positively increase the release of eDNA during biofilm development [59]. Emodin, an anthraquinone derivative obtained from *Polygonum cuspidatum* and *Rheum palmatum*, decreased *S. aureus* biofilm growth by intervening in the release of eDNA and downregulating the expression *cidA* [60]. Thus, eDNA could be a target for anti-biofilm agent screening.

As recently reported, Z-form eDNA accumulates as biofilm matures, and the compounds driving Z-DNA into B-DNA, such as chloroquine, were found to remarkedly disrupt extant biofilm EPS [61]. Universally, the two-member DNABII family of proteins acts as linchpin proteins to stabilize the structure of eDNA required for the biofilm matrix's stability [62]. Therefore, targeted removal of DNABII proteins could result in rapid biofilm collapse, releasing resident bacteria more susceptible to antibiotics and host immune effectors [61,63].

### 3.4 Phenolsoluble modulins (PSMs)

PSMs, a class of small peptides with an *α*-helical structure and resulting surfactant-like characteristics, are produced by most staphylococcal species, especially *S. aureus* and *S. epidermidis* [64]. *S. aureus* secretes four shorter PSM*α* peptides (∼20 amino acids), two longer PSM*β* peptides (∼40 amino acids), and the RNAIII-encoded *δ*-toxin [65]. PSMs have been documented as determinants of *S. aureus* virulence [65] and potential peptides to stimulate inflammatory responses [66]. With the given surfactant-like properties, PSMs facilitate biofilm structuring to form channels which makes nutrition available to *S. aureus* in the deeper layer of the biofilm and promote biofilm detachment to free bacteria [67,68]. In *S. aureus*, PSMs are soluble and can aggregate into the amyloid fibers to stabilize the biofilm structure [69]. Sinomenine is a natural alkaloid isolated from *Sinomenium acutum* that significantly reduces *S. aureus* biofilm dispersal due to cell-cell adhesion, PIA, and PSMs production [70]. Hence, PSMs might represent a potential research target.

## 4 Promising targets in the regulation of *S. Aureus* biofilm formation

Biofilm formation includes herd behavior. Multiple regulatory systems strictly control each step, from the initial attachment to the maturation and dispersion of biofilm.

### 4.1 Quorum ensing(QS) system

QS system is an internal communication system of bacteria, where the expression of relevant genes is initiated when the changes in the signal molecules reach a certain threshold. It involves multiple signal transduction pathways that regulate biofilm formation, virulence, motility, and sporulation [48]. In *S. aureus*, the research on QS system mainly focused on the accessory regulatory factor (Agr) system and LuxS/autoinducer-2 (AI-2) system [[71], [72], [73]].

**Fig. 2:**
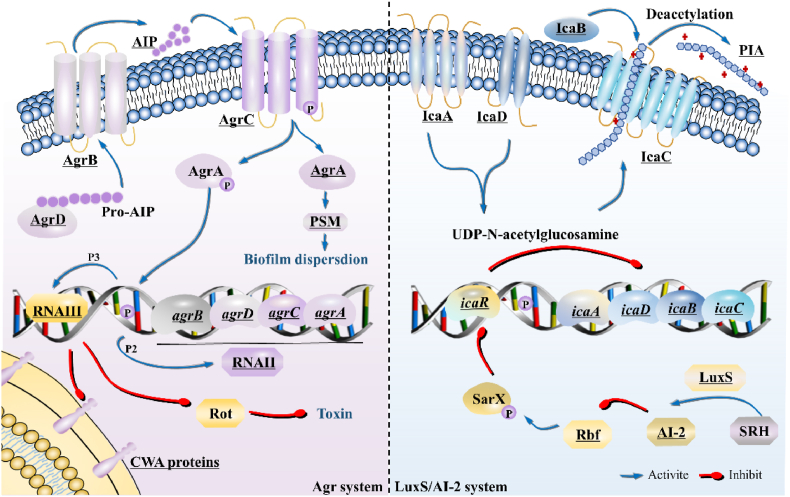
Mechanism of QS system for *S. aureus* biofilm regulation. In the Agr system, RNAII consists of *agrB/D/C/A* genes, encoding the AgrB/D/C/A proteins required for the biosynthesis, transport, and target gene regulation of AIP. RNAIII, as an essential effector, upregulate genes encoding exoproteins and downregulate genes encoding surface adhesins. In the LuxS/AI-2 system, LuxS catalyzes the synthesis of AI-2, inhibits Rbf and promotes *icaR* expression to repress IcaA/D/B/C, ultimately reducing the formation of PIA-dependent biofilms. QS, Quorum sensing; Agr, accessory gene regulator protein locus; AIP, auto-inducing peptide; PSM, phenol-soluble modulin; LuxS, S-ribosylhomocysteine lyase; AI-2, interspecies autoinducer. Key molecules of potential antibiofilm targets are underlined.

The Agr system in *S. aureus* (Fig. 2) contains RNAII and RNAIII, activated by the promoters P2 and P3, respectively [74]. The RNAII consists of *agrB/D/C/A* genes, encoding AgrB/D/C/A proteins required for auto-inducing peptide (AIP) biosynthesis, transport, and regulation of target genes [75]. AgrD, the precursor of AIP, is synthesized and exported to the plasma membrane by a feat of AgrB [76]. The accumulated AIP then binds to and activates the histidine kinase AgrC, leading to autophosphorylation and activating the signal transduction process, and eventually inducing a positive feedback loop on P2 and P3 promoters [77]. In addition, the elevated AIP can promote biofilms to depolymerize by increasing the secretion of extracellular protease [48]. RNAIII, as an important effector, upregulates genes encoding exoproteins, including haemolysins, toxins, and exoproteases, and downregulates genes encoding surface adhesins, such as FnBPs and serine-aspartate repeat family proteins [78,79]. Activation of the Agr system could be considered as an attractive antibiofilm strategy since it does not only inhibit the biofilm formation but also disperse the biofilm. Brazilin, a flavonoid from *Caesalpinia sappan*, has also been reported to inhibit biofilm formation by manipulating the Agr-related function [80]. However, Agr system can positively increase virulence factors, providing a mechanism for bacteria to rapidly adapt to changing environmental conditions. Hence, precisely manipulating the Agr system to inhibit biofilm formation without increasing virulence poses a great challenge. As practical application of this strategy would turn on the autoinduction of all four Agr subgroups, a promising reagent could be a cocktail of “clean” activators, each of which stimulates one or more agr variants without extensively affecting the rest [77]. The activation of AgrC involves an impediment to an intrasteric inhibitory docking interaction, providing a potential strategy for inhibiting biofilms by activating AgrC [81].

The S-ribosylhomocysteine lyase (LuxS)/AI-2 system (Fig. 2) regulates the formation of PIA-dependent biofilms by modulating the transcriptional regulation of intercellular adhesin (*ica*) locus [82]. Specifically, PIA is synthesized via proteins IcaA/D/B/C. IcaA and IcaD synergistically synthesize UDP-N-acetylglucosamine, followed by being exported through IcaC. Afterward, IcaB regulates the partial deacetylation of PIA to increase positive charge, thus improving adhesion [39]. LuxS can catalyze the biosynthesis of AI-2 and suppress the expression of *rbf*, thus promoting the expression of *icaR*, reducing the transcription level of *icaA*, and reducing PIA-dependent biofilm formation [72,83]. Paeoniflorin, a monoterpenoid glycoside extracted from *Paeoniaceae* plants such as peony, can reduce the luxS/AI-2 system-controlled biofilm formation and virulence [84]. Biosurfactants isolated from *Pediococcus acidilactici* and *Lactobacillus plantarum* were found to interfere with the release of AI-2 [11]. Thus, the LuxS/AI-2 QS system can be a competitive target in intercellular communication.

### 4.2 Two-component signal transduction systems (TCSs)

TCSs comprise two components, i.e., (i) sensory histidine kinase (HK), which senses the external environmental stimuli, and (ii) response regulator (RR), which regulates downstream target gene expression and quickly enhances the adaptive viability of bacteria [85]. Besides the Agr system mentioned above, TCSs in *S. aureus,* such as the YycFG and SaeRS systems, have emerged as novel targets against biofilms [86,87].

YycFG, also known as VicRK or WalRK, is the only essential two-component regulator contributing to bacterial pathogenicity and biofilm formation in *S. aureus* [88]. YycF was reported to have the ability to directly regulate the predicted promoter regions of the biofilm formation-related genes (e.g., *sarA* and *icaA*), and antisense yycG RNA (ASyycG) strategy successfully inhibited the transcription of *sarA* and *icaA*, thus decreasing the biofilm amass [86]. In *S. aureus*, YycFG also controls the autolysin synthesis related to cell wall metabolism and biofilm formation [89]. Overall, these data imply that inhibition of YycFG could reduce biofilm formation and bacterial pathogenicity, providing a promising target for the management of *S. aureus* infections. Rhodomyrtone, isolated from *Rhodomyrtus tomentosa*, has been speculated to interfere with the YycFG system, hence suppressing the expression of MRSA on the secreted proteins such as exoenzymes and antigenic proteins [90].

SaeRS has an important role in the regulation of *α*-toxin, *β*-haemolysin, staphylococcal immunoglobulin-binding protein, leucocidin, and toxic shock syndrome toxin-1, etc. [91]. In SaeRS TCS, the HK SaeS controls the expression of several exoproteins, including *α*-hemolysin and FnBPs [87], which is a potential therapeutic target during *S. aureus* biofilm formation. Xanthoangelol B, a prenylated chalcone obtained from *Angelica keiskei Koidzumi*, was found to bind directly to SaeS and inhibit its histidine kinase activity, demonstrating a possibility of a broad-spectrum inhibitor of histidine kinases [92].

### 4.3 SarA family proteins

The formation of *S. aureus* biofilm is also controlled by SarA family proteins, mainly including SarA, Rot, and MgrA. SarA can directly upregulate the expression of exoproteins and interconnect with the Agr system, where it can repress the production of extracellular proteases during the biofilm formation [93]. As a transcriptional activator, SarA enhances the transcription of *ica* operon and the production of PIA precursor to activate biofilm development [93]. Rot, an important regulator of virulence and biofilm-related genes expression in *S. aureus*, functions as a positive regulator of ClfB, SdrC, and SarS, which increases the surface protein level and decreases the extracellular enzyme level [94]. In *S. aureus*, RNAIII can regulate the synthesis of Rot via blocking its translation [95]. MgrA acts as a negative regulator against biofilm formation by repressing the production of adhesins [96]. (+)-Nootkatone treatments were found to down-regulate the expression of the *sarA* gene, thereby decreasing the PIA production and biofilm formation [43].

### 4.4 Alternative sigma factor σ^B^ (SigB)

SigB can promote the initial biofilm formation and repress the biofilm dispersal. Additionally, SigB indirectly regulates the biofilm formation by influencing other regulatory systems, e.g., by repressing RNAIII and SaeRS TCS, and upregulating the *sarA* expression according to the corresponding environments [27]. A number of natural products have been found to manipulate SigB to inhibit biofilm formation. For example, *Ginkgo biloba* exocarp extract was reported to downregulate the expression of the MRSA biofilm-associated factors *sarA* and *sigB* [97]. Similarly, *Scrophularia ningpoensis* honey can down-regulate the expression of genes *icaA*, *icaD*, *sarA*, *agrA*, and *sigB* in *S. aureus* [98].

## 5 Emerging natural products-based therapeutics against *S. aureus* biofilm

**Fig. 3:**
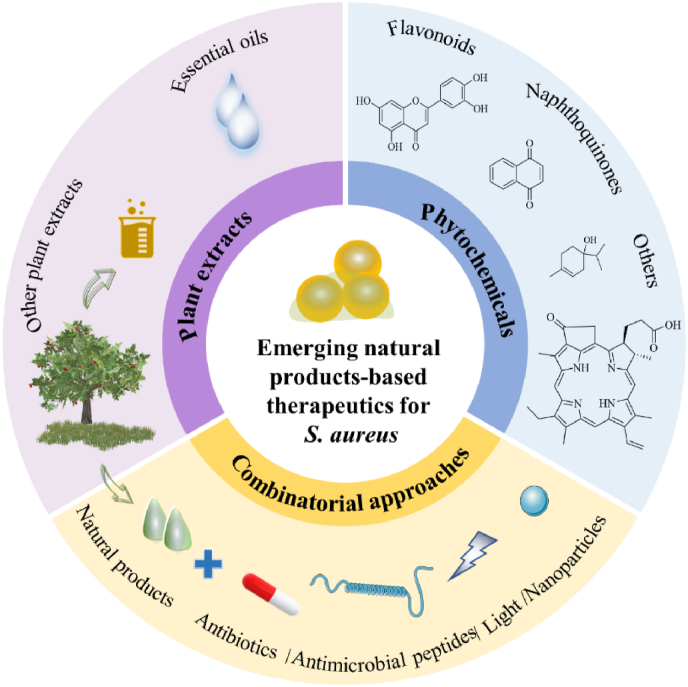
Novel natural products-based therapeutics for *S. aureus*.

**Fig. 4:**
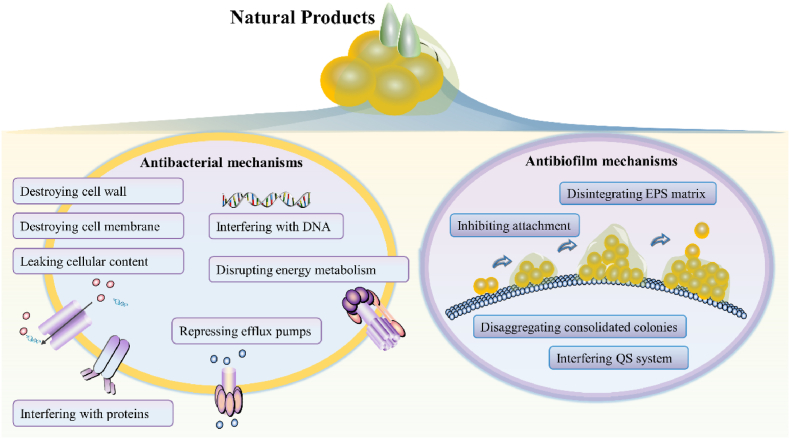
Antibacterial and antibiofilm mechanisms of natural products.

Natural products have represented powerful therapeutics against pathogens since the golden age of antibiotics in the mid-20th century [99]. Since combinatorial approaches have yielded effective drugs, developing new antibiofilm agents around different natural products (Fig. 3), such as plant extracts (Table 1) and phytochemicals (Table 2), is likely to lead to promising new strategies to combat biofilm. The antibiofilm activity of natural compounds primarily lies in certain aspects, including inhibition of bacterial cell attachment and adhesion, suppression of polymer (e.g., ECM) formation, reduction in the generation of pathogenic factors, and interruption of QS system. The current antibacterial and antibiofilm mechanisms of natural products are summarized in Fig. 4. To be note, although natural products have long been considered as nontoxic, on many occasions they have not been as safe as commonly thought [100,101]. Hence, it's definitely needed to study its toxicity before a natural product being nominated as a high-priority candidate in clinical treatments.

**Table 1:** Recently emerging plant extracts with antibiofilm activity.

Source	Extract	Strain	MIC (*μ*g/mL)	Biofilm formation inhibition	Mature biofilm disruption	Target	*In vivo*	Ref.
*Acacia macrostachya*	Stem barks, MeOH	*S. aureus*	>500	>60 %, 3.9 μg/mL	NS	QS	N	[118]
*Allium subhirsutum*	Bulbs, H_2_O	*S. aureus*	10000	56.21 %, 10 mg/mL	NS	QS	N	[116]
*Aphanamixis polystachya*	Leaves, DCM, MeOH	MRSA	6700	>40 %, 125 μg/mL	>20 %, 125 μg/mL	NS	N	[115]
*Artocarpus heterophyllus*	Heartwood, EtOH	MRSA	31.2–62.5	60 %, 31.2 μg/mL	NS	NS	N	[134]
*Azadirachta indica*	Leaves, EtOH	MRSA	1000	78.2–91.8 %, 1∼32 mg/mL	NS	NS	N	[113]
*Capsicum annuum*	Fruits, MeOH	*S. aureus*	64	53.8 %, 64 μg/mL	NS	NS	Y	[114]
*Catharanthus roseus*	Flowers, EtOH	MRSA	1000	54.5–99.7 %, 1∼32 mg/mL	NS	NS	N	[113]
*Cistus laurifolius, C. monspeliensis, C. parviflorus, C. salviifolius*	Leaves, EtOH	*S. aureus*	128∼256	NS	NS	NS	N	[111]
*Crithmum maritimum*	Leaves, EtOH	*S. aureus*	NS	82 %, 1 mg/mL	NS	NS	N	[112]
*Croton blanchetianus*	Leaves, H_2_O	MRSA	780	100 %, 780 μg/mL	NS	NS	N	[103]
*Croton conduplicatus*	Leaves, H_2_O	MRSA	512	22 %, 512 μg/mL	27 %, 512 μg/mL	NS	N	[104]
*Cuminum cyminum*	Seeds, H_2_O	MDR *S. aureus*	1.25–5	NS	NS	QS	N	[105]
*Eruca sativa*	Plants, EtOH	*S. aureus*	125	67.5 %, 125 μg/mL	73.45 %, 125 μg/mL	EPS; SrtA	N	[121]
*Glycyrrhiza glabra*	Plants, EtOH	*S. aureus*	NS	NS	99.7 %, 400 μg/mL	NS	N	[137]
*Gmelina arborea*	Leaves, H_2_O	*S. aureus*	90	46 %, 1 mg/mL	NS	NS	N	[149]
*Illicium verum*	Fruits, MeOH	MDR *S. aureus*	4800	74 %, 2.4 mg/mL	NS	AgrA, SarA	N	[119]
*Melia azedarach*	Leaves, DCM, MeOH	MRSA	2420	>50 %, 125 μg/mL	>60 %, 125 μg/mL	NS	N	[115]
*Myrsine umbellata*	Leaves, H_2_O, EtOH	*S. aureus*	1250	NS	84.28 %, 1.25 mg/mL	NS	N	[106]
*Origanum vulgare*	Commercial	*S. aureus*	48	>40 %, 6 μg/mL	54.05 %, 192 μg/mL	QS	N	[107]
*Polyalthia longifolia*	Leaves, MeOH, EtOH	MRSA	39∼78	99.5 %, 39 μg/mL	NS	NS	N	[135]
*Viburnum opulus*	Fruits/barks, acetone, EtOH	*S. aureus*	≥1000	∼20.4 %, 100 μg/mL	NS	SrtA; SpA	N	[120]

N, no; Y, yes; NS, not specified; DCM, dichloromethane; MeOH, methanol; EtOH, ethanol; MIC, minimum inhibitory concentration; MRSA: methicillin-resistant *S. aureus*; MDR, multidrug-resistant.

**Table 2:** Recently emerging natural compounds with antibiofilm activity.

Compound	Structure	MIC (*μ*g/mL)	MBIC (*μ*g/mL)	Biofilm formation inhibition	Mature biofilm disruption	Target	*In vivo*	Ref.
1,4-NQ		≤100	NS	55 ± 2 %, 10 μg/mL		ROS	N	[128]
3-HBA		400	NS	62.69 %, 6.25 μg/mL	NS	AgrA, SarA	N	[119]
Biochanin A		64∼256	128∼512	39.8–58.2 %, 32 μg/mL	24.8–52.1 %, 128 μg/mL	EPS, *icaA, srtA, eno*	N	[42]
Glabridin		6.25	<50	NS	NS	NS	N	[137]
Kaempferol		512	512	70∼80 %, 64 μg/mL	35∼55 %, 128 μg/mL	*fnbpA, fnbpB*	N	[50]
Luteolin		32∼64	32	99.72 %, 64 μg/mL	88.22 %, 128 μg/mL	*fnbpA, fnbpB, icaA, clfA*	N	[50]
Menadione		64∼256	NS	>90 %, 64 μg/mL	>85 %, 1024 μg/mL	ROS	N	[129]
Nerolidol		1000	∼500	>70 %, 1 mg/mL	NS	NS	N	[131]
Pyropheophorbide A		NS	NS	NS	NS	SrtA	N	[121]
Quercetin		256	128	70∼80 %, 64 μg/mL	35∼55 %, 128 μg/mL	*fnbpA, fnbpB*	N	[50]
Shikonin		15.6	<15.6	∼50 %, 15.6 μg/mL	NS	*icaA, fnbpA, agrA, saeS, sigB*	N	[130]
Sinomenine		>73.2	NS	NS	26.29 %, 9.1 μg/mL	*agrA, icaA,* PIA, PSM	N	[70]
Terpinene-4-ol		48	NS	>40 %, 12 μg/mL	70.97 %, 192 μg/mL	QS	N	[107]

N, no; NS, not specified; MBIC, minimal biofilm inhibitory concentration.

### 5.1 Plant extracts

#### 5.1.1 Essential oils (EOs)

EOs, volatile compounds extracted from medicinal plants, have shown great therapeutic potential against microbial biofilms [[102], [103], [104], [105]]. The EOs extracted from *Croton* species (e.g., *C. blanchetianus* and *C. conduplicatus*) can inhibit biofilm formation and reduce preformed biofilms of MSSA and MRSA strains [103,104]. Saponins, alkaloids, flavonoids, free steroids, and tannins could be responsible for the eradication of biofilm, since they act by dehydrating the cell wall, disaggregating consolidated colonies, preventing nutrient replacement, and disintegrating the structure of mature biofilm [106]. Sharifi et al. further investigated the antibiofilm mechanism of *Cuminum cyminum* essential oil (CcEO) against multidrug-resistant (MDR) *S. aureus*. In the case of QS inhibitory potential, the CcEO at sub-MIC concentrations (0.625–1.25 μL/mL) significantly reduced the *hld* and *ica* expression by 3.13- and 2.33-fold, respectively [105]. In addition, *Origanum vulgare* essential oil at a concentration of 1/8 MIC (6 μg/mL) exerted an anti-attachment effect against *S. aureus*, while the effect of terpinene-4-ol within this EO was observed at 1/4 MIC [107]. It has been demonstrated that using EOs to inhibit pathogenic bacteria attachment is a strategic way to prevent biofilm formation. Meanwhile, the *O. vulgare* EO and terpinene-4-ol at the concentrations of MIC∼4 MIC eradicated the mature biofilms by 10.36–54.05 % and 62.28–70.97 %, respectively [107]. Therefore, *O. vulgare* EO was more effective against *S. aureus* biofilm formulation, while terpinene-4-ol was superior in eradicating mature biofilms. Additionally, *O. vulgare* EO presented better anti-QS activity than terpinene-4-ol in a concentration-dependent manner [107]. Overall, the synergistic effects between various compounds in EOs may lead to the interference of the QS system and subsequently affect the ability to develop biofilms and generate virulence factors in bacteria. The superiority of EOs may also originate from the non-polar components with high diffusion coefficients that easily penetrate the biofilm and damage membrane fluidity [108]. Noteworthy, EOs have a low potential to develop microbial resistance due to multiple targets [109] and the inhibition of the function of efflux pumps [110]. Therefore, EOs can be used in combination with antibiotics since they mediate the re-sensitization of the bacteria to some drugs, which helps address the problem of antibiotic resistance.

#### 5.1.2 Other plant extracts

The hydroethanolic extracts of numerous plants have been reported to significantly reduce the *S. aureus* biofilm formation, which could be explained by the presence of chlorogenic, quercetin, and rutin [[111], [112], [113]]. Likewise, due to rich and diverse phytochemical compositions, the methanolic extract of *Capsicum annuum* at 64 μg/mL showed an inhibition rate of 53.8 % against *S. aureus* biofilm, comparable to amoxicillin [114]. *Aphanamixis polystachya* and *Melia azedarach* extracts at sub-lethal concentrations were found to inhibit and disrupt the biofilms formed by MRSA, residing in the existence of limonoids, phenolics, and oxygenated triterpenoids [115]. Recently, researchers have been exploring the mechanism of plant extracts against biofilms. For instance, *Allium subhirsutum* aqueous extract proved to interrupt QS system against a diverse panel of microorganisms. By using gas chromatography-mass spectrometry, the potential bioactive compounds were identified as 5-hydroxymethylfurfural, methyl methanethiolsulfonate, furfural, trisulfide, di-2-propenyl, and diallyl disulfide [116]. In particular, 5-hydroxymethylfurfural at 125 μg/mL have been previously reported to inhibit the *S. aureus* biofilm formation by 82 %, which might be the contributing component of *A. subhirsutum* [117]. The possible quenching of QS mechanism was also demonstrated in the crude extract of *Acacia macrostachya* [118]. In addition, the methanolic extract of *Illicium verum* showed a biofilm inhibition effect with 3-hydroxybenzoic acid (3-HBA) as the primary active constituent, which strongly interacted with the active site residues of SarA and AgrA of *S. aureus* [119]. Moreover, the inhibition of SrtA and staphylococcal protein A (SpA) expression, as well as EPS production, are emerging targets of extracts from plants such as *Viburnum opulus* [120] and *Eruca sativa* [121].

### 5.2 Phytochemicals

#### 5.2.1 Flavonoids

Novel natural antibiofilm agents comprise phenolics, terpenoids, alkaloids, polypeptides, lectins, and polyacetylenes [122]. Flavonoids, an important class of phenolics, are well known as antibacterial agents against a wide range of pathogenic microorganism through inhibition of the cell attachment and biofilm formation, alteration of the membrane permeability, inhibition of nucleic acid synthesis, inhibition of energy metabolism, inhibition of the porin on the cell membrane, inhibition of cytoplasmic membrane function, and attenuation of the pathogenicity [123]. Luteolin, quercetin, and kaempferol are common flavonoids found in medical plants, fruits, and vegetables. Quercetin has been found to remarkably reduce the production of elastase, protease and pyocyanin in *Pseudomonas aeruginosa*, violacein in *Chromobacterium violaceum*, as well as EPS in *Yersinia enterocolitica*, inhibiting biofilm formation and disrupting the preformed biofilm [124,125]. Recently, the expression levels of biofilm-related genes in *S. aureus* were found to be decreased by luteolin, quercetin, and kaempferol at concentrations of 8∼128 μg/mL. In conjugation with luteolin, quercetin, and kaempferol can elicit a more effective antibiofilm effect by synergistically down-regulating *fnbpA* and *fnbpB* expression [50]. Another study also revealed that upon luteolin treatment, the expression of key genes involved in virulence (*hla*) and biofilm formation (*icaA*, *fnbpA*, and *clfA*) was downregulated in the wild-type *S. aureus* strain [126]. The inefficacy of luteolin with respect to the virulence factor in the *agr* mutant strain suggested the *agr*-mediated anti-virulence and antibiofilm potential of flavonoids.

#### 5.2.2 Naphthoquinones

Among various antimicrobial agents, naphthoquinones and their derivatives have received great attention due to their exceptional structural diversity and varied biological properties [127]. 1,4-Naphthoquinone (1,4-NQ) at a concentration of 10 μg/mL was found to inhibit the *S. aureus* biofilm formation by 55 %, where the microbial motility was reduced. Besides, 1,4-NQ increased the cellular accumulation of reactive oxygen species (ROS), which could impact biofilm formation [128]. In their study, Mone et al. reported that menadione, an analogue of 1,4-NQ, was effective against different MDR strains of *S. aureus* not only by inhibiting biofilm formation (>90 % at MICs ranging from 64 to 256 μg/mL) but also by removing preformed biofilms (>85 % at 1024 μg/mL), accompanied by enhanced levels of ROS [129]. Likewise, shikonin (SKN), a highly liposoluble 1,4-NQ derivative extracted from the root of *Lithospermum erythrorhizon*, was found to inhibit the biofilm formation of clinical MRSA strains at concentrations lower than MIC (15.6 μg/mL). Further mechanism studies showed that SKN interferes with the expression of *icaA* and *fnbpA*, indicating that SKN could inhibit cell attachment during biofilm formation. In addition, SKN at sub-inhibitory concentrations down-regulated the expression of virulence factor regulatory genes (*agrA, saeS,* and *sigB*), subsequently affecting the transcription of exoprotein-coding genes (*hla* and *sea*), and further elucidating the relevance of SKN-induced reduction of MRSA virulence factors [130]. Overall, naphthoquinones possessing antibacterial and antibiofilm activities are imperative in the era of drug-resistance developed by bacterial pathogens.

#### 5.2.3 Other natural compounds

Alkaloids, aromatic acids, and sesquiterpenes are natural compounds with antibiofilm activities against *S. aureus*. For example, the alkaloid sinomenine can significantly upregulate *agrA* expression and down-regulate *icaA* level [70]. Aromatic acids such as 3-HBA inhibit *S. aureus* biofilm activity targeting the Agr and SarA systems [119]. Nerolidol, a sesquiterpene, was found to inhibit *S. aureus* biofilm by > 70 % at concentrations ranging from 1 to 4 mg/mL [131].

### 5.3 Combinatorial approaches

#### 5.3.1 Combination of different compounds

Due to limited therapeutic options for *S. aureus*, identifying effective combinations provides an alternative for infection treatment. For instance, besides having significant biological activity, curcumin-based metal complexes enhance the bioavailability of curcumin. At the concentration of 100 μM, curcumin inhibited *S. aureus* biofilm formation (55.8 %), whereas oxovanadium complex of curcumin has a significantly stronger effect (82 %), which could reside in the synergistic effect of complex mechanisms, including alkaline phosphatase inhibition and antibacterial mechanism [132].

The antibiofilm effects of combinational terpenes including (−)-*trans*-caryophyllene, (*S*)-*cis*-verbenol, (*S*)-(−)-limonene, (*R*)-(+)-limonene, and linalool were also evaluated. As shown in the results, all combinations of terpenes inhibited biofilm formation by > 50 % without affecting bacterial growth. Of note, (−)-*trans*-caryophyllene and linalool at 500 μg/mL for each acquired the most superior effect with an inhibition percentage of 88 %, resulting from the down-regulation of *sdrD*, *spa*, *agr*, and *hld* genes related to cell adhesion and QS system, as well as the up-regulation of *cap5B* and *cap5C* genes associated with the production of capsular polysaccharides [133]. Taking advantage of different mechanisms, the combinations are expected to achieve enhanced effects, thus overcoming the resistance of *S. aureus*.

#### 5.3.2 Combination of plant extract and antibiotic

Because pure antibiotic or natural product therapy might be insufficient to combat drug-resistant bacterial infections, combining plant extract and antibiotics is a potential strategy for improving efficacy. Bazmi et al. demonstrated that the combination of artocarpin-rich *Artocarpus heterophyllus* extract with ampicillin synergistically altered the membrane permeability of MRSA, which led to the release of intracellular materials. Moreover, the tested components revealed that high-level suppression of biofilm formation (62–76 %) at their MICs, which could potentiate the antibacterial effects of each other at their sub-inhibitory concentrations (1/2∼1/16 MIC) in the synergistic cocktails by increasing the penetration of compounds into the matrix of biofilm [134]. Likewise, *Polyalthia longifolia* leaf extracts showed a synergistic action with penicillin against a clinical MRSA isolate and significantly inhibited the biofilm formation, implying their combinational antibiofilm potential [135]. Sannat et al. explicitly reported that the methanolic extract of *Hemidesmus indicus* root synergized the antibiofilm activity of amoxicillin and clindamycin against MRSA isolates. Moreover, in the kidney and liver of MRSA-infected mice, the combinations significantly reduced bacterial load, disease activity score, and Gram-positive spots [136]. Therefore, the combination of plant extracts with antibiotics is advocated in the treatment of biofilm-associated infections. In particular, the bioactive compound-rich plant extracts can be used in place of pure compounds in pharmaceutical industries, given the lower cost of production.

#### 5.3.3 Combination of the natural compound and antimicrobial peptide

The ethanol extract of *Glycyrrhiza glabra* showed potent biofilm eradication activity against *S. aureus* through glabridin with the minimum biofilm eradication concentration of 50 μg/mL. Furthermore, glabridin combined with the antimicrobial peptide *ɛ*-poly-*l*-lysine resulted in broad, more potent biofilm eradication activities. The synergistic effect was speculated to occur due to the enhanced permeability of microbial cell membrane in biofilms by *ɛ*-poly-*l*-lysine, allowing more glabridin to transport into the cell, thus generating ROS and causing oxidative damage to cellular structure, lipids, proteins, and DNA [137].

#### 5.3.4 Combination of the natural compound and photodynamic therapy

As a nonantibiotic microbicidal technology, photodynamic therapy relying on blue light has been extensively studied to disinfect various bacteria, including MRSA [138]. Lu et al. reported that blue light and carvacrol synergistically killed various bacteria, including planktonic cells, biofilm cells, and per-sisters. Carvacrol at 0.2 mg/mL and blue light 450 nm at 75 J/cm^2^ decreased the thickness of *in vitro* MRSA biofilm from 32.4 μm to 1.7 μm and completely or substantially cured both acute and established MRSA biofilm-associated infections of full thickness murine third-degree burn wounds. Further mechanistic studies showed that carvacrol was photocatalyzed to a series of photoreactive substrates, which underwent photolysis or additional photosensitization reactions and thus generated robust cytotoxic ROS [139]. Recently, Sharma et al. used alginate microfibers as a carrier of curcumin, followed by being irradiated with blue light (450 nm) to assess the efficacy of the combined therapy against MRSA. The curcumin-loaded microfibers exhibited great potential when combined with photodynamic therapy, which resulted in the complete eradication of the MRSA biofilms [140]. These results suggested that the combination of natural products and photodynamic therapy is a potent tool against biofilm-forming bacteria that can reduce their antimicrobial resistance.

#### 5.3.5 Combination based on natural compound and nanoparticle

The rapid development of nanoparticles (NPs) in drug delivery systems has led to many studies exploring the antibacterial and antibiofilm effects of NPs and the potential for resistance [[141], [142], [143], [144], [145]].

Cerium oxide (CeO_2_) NPs prepared from aqueous leaf extract of *Pometia pinnata* at 512 μg/mL showed inhibition on *S. aureus* biofilm by 73 % [146]. However, in the case of Zr/Sn-dual doped CeO_2_ NPs, only the 10 % Zr/Sn-dual doped sample showed *S. aureus* biofilm inhibition at 512 μg/mL, whereas the lower concentration of dual doped NPs did not respond to antibiofilm activity [147]. Despite the improvement of transition metal ion dopants on the catalytic and photocatalytic oxidation activity of pure CeO_2_ NPs, it unexpectedly reduced the antibiofilm activity. Therefore, the oxidative stress and cellular toxicity resulting from the Ce^3+^ release and ROS generation were proposed as the antibiofilm mechanisms of CeO_2_ nanoparticles [146], similar to the current antibacterial mechanisms of metallic NPs, such as metal ion release and oxidative stress [148].

Phyto-fabrication of silver nanoparticles (AgNPs) using *Gmelina arborea* (GA) extract was also studied, where the biofilm inhibition by aqueous leaf extract of GA at 1000 μg/mL was 46 %. GA-AgNPs were more effective than GA-extract, and their antibiofilm activity increased further when loaded on hydrogel as GA-AgNPs-PF127 (59 %), making it a novel distinguishing feature. The acidic-basic affinity between Ag^+^ and sulfur-containing proteins and phosphorous moieties of DNA can enhance bactericidal capability. Besides, the ability of AgNPs to perturb the bacterial cells could also be due to their atomicity. The smaller the size, the higher the magnitude. GA-AgNPs in size range of 34∼40 nm own high surface area to volume ratio, explaining their stronger inhibitory effect than GA-extract. Furthermore, the synergy between NPs and phytochemicals forms target site-oriented phyto-NPs that disrupt the membrane at the interaction site [149]. Likewise, AgNPs produced from *Cedecea* and *Anthemis* species successfully inhibited the biofilms of different pathogens including *S. aureus*, *S. epidermidis*, *Escherichia coli* and *Pseudomonas aeruginosa* [150,151].

Sonodynamic therapy is a noninvasive and effective therapeutic strategy, which has broadened the way toward dealing with bacteria and biofilms. It uses a combination of ultrasound waves, molecular oxygen, and a sonosensitizer to generate cytotoxic ROS, thereby leading to the death of target microbial cells [152]. Pourhajibagher et al. accessed the combined antibiofilm efficacy of nano-emodin (N-EMO) and sonodynamic therapy against multi-species bacterial biofilms, including *S. aureus, Acinetobacter baumannii*, and *Pseudomonas aeruginosa*. As the results demonstrated, there were 71.0 % and 81.5 % reductions in the mixed biofilms following sonodynamic therapy using 0.39 μg/mL and 0.75 μg/mL of N-EMO, respectively. Furthermore, N-EMO-mediated sonodynamic therapy significantly downregulated the expression of *agrA* by 3.6-fold [153], highlighting the potential of NPs-mediated sonodynamic therapy in antibiofilm and reduction of virulence factors associated with biofilms. Broad spectrum inhibition of bacterial growth and great antibiofilm effect by NPs make them pertinent advocate for curing infectious diseases.

## 6 Conclusions

Either the biofilm formation inhibition or preformed biofilm disruption could be very effective in the face of bacterial infection. The antibiofilm agents formed by natural products, such as plant extracts and phytochemicals, are promising candidates for treating chronic and persistent biofilm infections. The research on natural products-based antibiofilm agents has been focusing on (i) inhibiting biofilm formation or degrading mature biofilm that targets PIA, eDNA, and proteins; and (ii) interfering with the regulation network of biofilm development that targets the QS system. Natural antibiofilm compounds can inhibit initial bacterial adhesion and/or downregulate the expression of biofilm-related genes, with higher reliability in structure and function than traditional antibacterial agents. On the one hand, an anti-adhesion method can be used as a unique antibiofilm strategy, thus future studies on eradicating biofilms by repressing the adhesion proteins may provide a new dimension for the research and development of antibiofilm agents. On the other hand, different phytochemicals can effectively inhibit the expression of genes associated with biofilm formation or regulation, and further research in this area may prove to be a better way to treat biofilm-associated infections. The current studies revealed that flavonoids can regulate the expression of genes including *clfA*, *fnbpA*, *fnbpB*, *icaA*, *saeS*, *sigB*, *srtA*, and *eno*, and degrade EPS matrix, while alkaloids modulate PIA and PSM more, terpenes target QS, as well as aromatic acids interfere with AgrA and SarA (Table 2). In addition, the continuous advances in nanomaterials have also facilitated the development of antibiofilm agents that inhibit the development of resistance by physically destroying bacterial cell membranes and biofilm matrix.

In conclusion, this preview provides valuable information for screening promising targets for antibiofilm agents, while the active compounds/combinations provide attractive candidates for fighting against *S. aureus* infections. Although some antibiofilm natural products have been identified with preliminarily elucidated mechanisms of action, most of these studies were observational, strongly requiring in-depth exploration of relevant mechanisms. Moreover, the available data on natural products against *S. aureus* biofilms are generally limited to *in vitro* studies (Table 1, Table 2), calling for urgently developing *in vivo* models that can mimic the biofilm in human infections. Future novel and effective antibiofilm therapies will likely include more synergistic actions based on natural products that exhibit antibiofilm properties.

## Compliance with ethics requirements

This article does not contain any studies with human or animal subjects.

## CRediT authorship contribution statement

**Xiying Wu:** Writing – review & editing, Writing – original draft, Methodology, Investigation, Conceptualization. **Huan Wang:** Writing – review & editing, Methodology. **Juan Xiong:** Writing – review & editing, Methodology. **Guo-Xun Yang:** Visualization. **Jin-Feng Hu:** Writing – review & editing, Supervision. **Quangang Zhu:** Writing – review & editing, Supervision, Conceptualization. **Zhongjian Chen:** Writing – review & editing, Supervision, Conceptualization.

## Declaration of competing interest

The authors declare that they have no known competing financial interests or personal relationships that could have appeared to influence the work reported in this paper.

## Data Availability

Data will be made available on request.
